# Cationic Carbosilane Dendrimers Prevent Abnormal α-Synuclein Accumulation in Parkinson’s Disease Patient-Specific Dopamine Neurons

**DOI:** 10.1021/acs.biomac.1c00884

**Published:** 2021-10-06

**Authors:** Raquel Ferrer-Lorente, Tania Lozano-Cruz, Irene Fernández-Carasa, Katarzyna Miłowska, Francisco Javier de la Mata, Maria Bryszewska, Antonella Consiglio, Paula Ortega, Rafael Gómez, Angel Raya

**Affiliations:** † Regenerative Medicine Program, and Program for Clinical Translation of Regenerative Medicine in Catalonia—P-CMR[C], L’Hospitalet de Llobregat (Barcelona), Institut d’Investigació Biomèdica de Bellvitge—IDIBELL, Barcelona 08907, Spain; ‡ Networking Research Center on Bioengineering, Biomaterials and Nanomedicine (CIBER-BBN), Madrid 28029, Spain; § University of Alcalá, Department of Organic Chemistry and Inorganic Chemistry and Research Institute in Chemistry “Andrés M. del Río” (IQAR), Madrid 28805, Spain; ∥ Department of Pathology and Experimental Therapeutics, Hospitalet de Llobregat (Barcelona), Universitat de Barcelona and Institut d’Investigació Biomèdica de Bellvitge—IDIBELL, Barcelona 08907, Spain; ⊥ Department of General Biophysics, Faculty of Biology and Environmental Protection, University of Lodz, Pomorska 141/143, Lodz 90-236, Poland; # Department of Molecular and Translational Medicine, University of Brescia, Brescia 25121, Italy; ¶ Institució Catalana de Recerca i Estudis Avançats (ICREA), Barcelona 08907, Spain

## Abstract

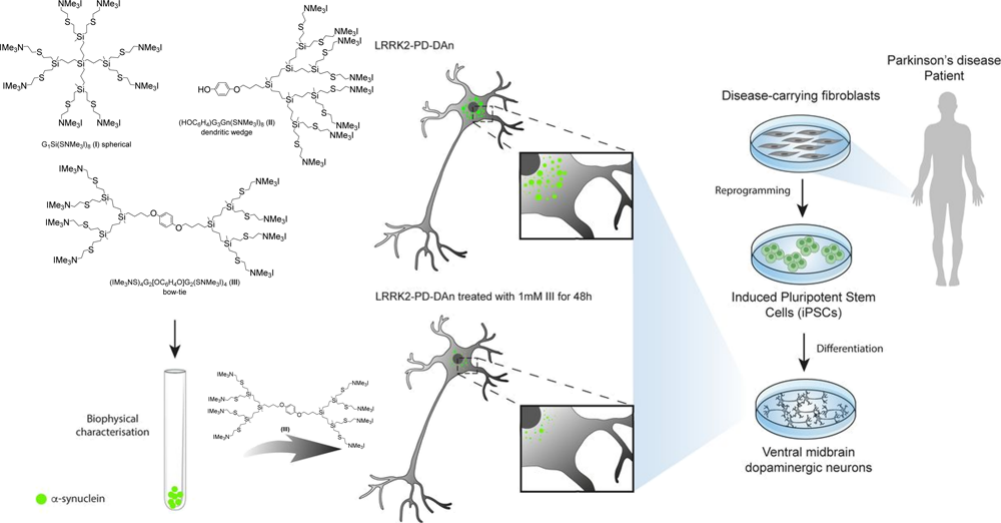

Accumulation of misfolded α-synuclein (α-syn) is a hallmark of Parkinson’s disease (PD) thought to play important roles in the pathophysiology of the disease. Dendritic systems, able to modulate the folding of proteins, have emerged as promising new therapeutic strategies for PD treatment. Dendrimers have been shown to be effective at inhibiting α-syn aggregation in cell-free systems and in cell lines. Here, we set out to investigate the effects of dendrimers on endogenous α-syn accumulation in disease-relevant cell types from PD patients. For this purpose, we chose cationic carbosilane dendrimers of bow-tie topology based on their performance at inhibiting α-syn aggregation *in vitro*. Dopamine neurons were differentiated from induced pluripotent stem cell (iPSC) lines generated from PD patients carrying the *LRRK2*^*G2019S*^ mutation, which reportedly display abnormal accumulation of α-syn, and from healthy individuals as controls. Treatment of PD dopamine neurons with non-cytotoxic concentrations of dendrimers was effective at preventing abnormal accumulation and aggregation of α-syn. Our results in a genuinely human experimental model of PD highlight the therapeutic potential of dendritic systems and open the way to developing safe and efficient therapies for delaying or even halting PD progression.

## Introduction

Alpha-synuclein (α-syn) is a small presynaptic protein widely and abundantly expressed in neurons throughout the nervous system.^1^ Even though the normal functions of α-syn are not completely understood, abnormal accumulation in neurons has been found in a variety of neurological diseases collectively known as synucleinopathies,^2,3^ including Parkinson’s disease (PD) in both sporadic and familial cases.^4^ PD is an age-related, chronic, and progressive neurodegenerative disorder, characterized by loss of dopamine neurons (DAns) within the substantia nigra pars compacta, which contributes to the cardinal motor symptoms of the disease.^5,6^ A pathological hallmark of PD is the presence of α-syn-containing intraneuronal inclusions, known as Lewy bodies.^2^ Both the presence and intensity of Lewy bodies closely correlate with disease progression^7^ and are thus thought to play a pivotal role in PD pathophysiology.^8^ The facts that mutations or multiplications in the *SNCA* gene (encoding α-syn) cause familial PD^9^ and that experimental models of α-syn overexpression^10,11^ or artificially high loads of aggregated or preformed α-syn fibrils^12−14^ lead to synaptic dysfunction and neuron death indicate that increased α-syn expression, accumulation, or aggregation in DAns may be sufficient to promote nigral degeneration.

Despite intensive research, PD remains an incurable neurological condition. Currently available treatments are designed to compensate the loss of dopamine and dopaminergic function, but they are palliative and limited by side effects and lack of long-term efficacy.^15,16^ Unfortunately, current therapeutic strategies do not cure or block PD progression, emphasizing the critical need for finding new disease-modifying approaches that halt, slow, or delay disease progression. Considering its importance in PD pathophysiology, α-syn could be an interesting therapeutic target for this purpose, and inhibition of α-syn aggregation using protein-solubilizing drugs is a potential therapeutic strategy.

Nanotechnology has contributed to our understanding of PD pathogenesis by providing tools that promote the solubilization of α-syn fibers and prevent the formation of α-syn aggregates.^17^ Different nanomaterials have been explored for their usefulness as antiparkinsonian, antioxidant, and/or neuroprotective agents, including nanoparticles,^18^ dendrimers,^19^ and carbon nanotubes.^20^ Among them, dendrimers are especially interesting as they are highly branched polymers with well-defined geometry and unique properties that can be functionalized according to the specific application for which they are designed. A variety of dendritic systems of different natures and topologies have been tested in the context of neurodegenerative diseases, including PAMAM,^21^ polypropyleneimine dendrimers,^22^ glycodendrimers,^23^ and phosphorous^24^ or carbosilane^25^ dendrimers, and shown to have an effect on α-syn aggregation.^21,24,26,27^ Moreover, dendrimers have also been reported to inhibit the aggregation of insoluble forms of amyloid^28^ and prion^29^ peptides and to facilitate their degradation.^30,31^

For the present studies, we focused on carbosilane dendrimers as they have been proven to be effective in a variety of biomedical applications in a topology-dependent manner^25,32−36^ and non-toxic at concentrations below 5 μM when used as transfecting or anti-cancer agents.^37,38^ One of the main properties of carbosilane dendrimers is their inherent hydrophobicity, provided by their characteristic skeleton, which allows interaction with biological membranes in an efficient manner^39^ and the presence of a permanent number of charges. For this reason, low generations of carbosilane dendrimers are effective for most applications, unlike the case of other dendritic skeletons. Specifically, in the context of protein misfolding diseases, cationic carbosilane systems have shown their ability to inhibit rotenone-induced α-syn fibrillation in mouse hippocampal cells^40^ and amyloidogenic islet amyloid polypeptide (IAPP) aggregation in a mouse model of type II diabetes.^25^ In this work, we investigated how the topology of cationic carbosilane dendrimers (spherical, dendritic wedge, or bow-tie) could influence the interaction with α-syn to avoid aggregation. After biophysical characterization of the interaction between α-syn and the different dendritic systems, we selected the bow-tie topology for testing effectiveness in an induced pluripotent stem cell (iPSC)-based experimental model of PD. Our results show that non-cytotoxic concentrations of cationic carbosilane dendrimers are effective in preventing abnormal accumulation and aggregation of α-syn in dopamine neurons from PD patients.

## Materials and Methods

### Materials

The spherical dendrimer G_1_Si(SNMe_3_I)_8_ (**I**), dendritic wedge (HOC_6_H_4_)G_3_Gn(SNMe_3_I)_8_ (**II**), and bow-tie system (IMe_3_NS)_4_G_2_[OC_6_H_4_O]G_2_(SNMe_3_I)_4_ (**III**) were synthesized according to the previously described protocol.^25,41^ For zeta potential and circular dichroism (CD) spectrometry, α-syn was purchased from Sigma-Aldrich (USA). For all experiments, α-syn and dendrimers were dissolved in phosphate-buffered saline (1.9 mM NaH_2_PO_4_, 8.1 mM Na_2_HPO_4_, and pH = 7.4).

### Methods

#### Zeta Potential

The measurements of the zeta potential (electrokinetic potential) were performed with a Zetasizer Nano ZS from Malvern, which uses electrophoresis and LDV (laser doppler velocimetry) techniques to allow measurement of the electrophoretic mobility of the molecules in the solution. The zeta potential value was calculated directly from the Helmholtz–Smoluchowski equation using Malvern software.^42^ The measurements were performed at 37 °C with three repetitions. Increasing concentrations of the bow-tie dendrimer in a range of 0.25–25 μM were added to a 1 μM solution of α-syn, and the zeta potential was measured.

The analysis of possible changes in the zeta potential as a function of the molar ratio can be approximated by the number of dendrimer molecules that can attach to one molecule of α-synuclein. The number of binding centers is the point (X, O) corresponding to the point of intersection of two straight lines tangent to the curve of the zeta potential as a function of the molar ratio.

#### CD Spectrometry

The circular dichroism (CD) spectra (190–260 nm) were recorded for α-syn in the presence/absence of dendritic systems on a Jasco J-815 CD spectropolarimeter, in 5 mm-path length quartz cuvettes, with a wavelength step of 0.5 nm, a response time of 4 s, and a scan rate of 50 nm/min, maintained at 37 °C. Each spectrum was the average of three repetitions. α-Syn was used at a concentration of 1 μmol/L. The changes in the secondary structure of α-syn in the presence and absence of dendritic systems **I–III** were tested immediately (dendrimers/dendron concentrations: 0.5–5 μmol/L) and after 48 and 72 h (dendrimers/dendron concentrations: 2 and 4 μmol/L) of incubation at 37 °C. Dendrimers/dendron alone at the experiments’ concentrations did not produce any discernible features in their CD spectra and were used as blanks for α-syn–dendrimer complex spectra.

#### iPSC-Derived DAn Generation

The iPSC lines used in our study were previously generated and fully characterized.^43^ Specifically, we used iPSC generated from two patients carrying the G2019S mutation in the *LRRK2* gene (LRRK2-PD) and from two healthy age-matched controls (CTRL) (Table 1). The competent Spanish authorities (Commission on Guarantees concerning the Donation and Use of Human Tissues and Cells of the Carlos III National Institute of Health) approved the use of human iPSCs in this work. For DAn differentiation, iPSCs were transduced with LV.NES.LMX1A.GFP and processed as previously described.^44^ Embryoid bodies (EBs) generated from iPSC colonies were cultured in nonadherent dishes for 3 days in the presence of mTeSR medium (Stem Cell Technologies) (stage 1). At stage 2, EBs were cultured for 10 days in suspension in N2B27 medium [DMEM/F12 medium (Gibco), neurobasal medium (Gibco), 1% B27 (Gibco), 0.5% N2 (Gibco), 1% GlutaMAX (Gibco), and 1% penicillin/streptomycin] supplemented with 10 ng/mL FGF2 (PeproTech), 100 ng/mL FGF8 (PeproTech), and 100 ng/mL SHH (PeproTech). For DAn maturation, at stage 3, the neural precursor cells (NPCs) were co-cultured with PA6 stromal cells for 3 weeks in N2B27 medium supplemented with FGF8 and SHH. Finally, at stage 4, dopaminergic neurons generated were dissociated using Accutase (Merck), re-plated on Matrigel-coated dishes, and cultured for 1 week in N2B27 with FGF8 and SHH. Correct differentiation was judged by co-immunostaining with neuron-specific class III-β-tubulin (TUJ1), a widely used neuronal marker, and tyrosine hydroxylase (TH), the main neuronal marker for dopaminergic neurons.

**Table 1:** Summary of Healthy Controls and Patients Used in This Study

code	status	gender	age at biopsy	mutation
CTRL1	Control	M	66	no
CRTL2	Control	F	48	no
LRRK2-PD1	Parkinson’s disease	M	66	G2019S (LRRK2)
LRRK2-PD2	Parkinson’s disease	F	63	G2019S (LRRK2)

#### (IMe_3_NS)_4_G_2_[OC_6_H_4_O]G_2_(SNMe_3_I)_4_ (**III**) Treatment

(IMe_3_NS)_4_G_2_[OC_6_H_4_O]G_2_(SNMe_3_I)_4_ (**III**) was added to DAns at day 3 of stage 4 of differentiation for 48 h. Then, dendrimers were removed, and cells were incubated for a further 48 h with fresh media before analysis. To evaluate the effect of dendrimers on DAn survival, cells were treated with two different concentrations of **III**, 1 and 3 μM. The numbers of surviving DAns were calculated by counting tyrosine hydroxylase (TH)-positive neurons. For subsequent experiments, DAns were treated with 1 μM **III** for 48 h, stained, and α-syn accumulation was analyzed.

#### Immunofluorescence

Cells were fixed with 4% paraformaldehyde in PBS at room temperature for 15 min. Cells were blocked and permeabilized with TBS with low triton (0.01% Triton X-100) and 3% donkey serum for 2 h. Subsequently, cells were incubated for 48 h at 4 °C with the following primary antibodies: mouse anti-TUJ1 (1:500, T8660, Sigma), rabbit anti-TH (1:250, sc-14007, Santa Cruz), and mouse anti-α-syn (1:500, 610787, BD Biosciences). Samples were then incubated with secondary antibodies for 2 h at 37 °C: Alexa Fluor 647 anti-mouse IgG2a (1:100, A21241, Invitrogen), Cy3 anti-rabbit IgG (1:200, 711-165-152, Jackson), and Alexa Fluor 488 anti-mouse IgG1 (1:100, A21121, Invitrogen). To visualize nuclei, slides were stained with DAPI (1:5000, Invitrogen) and then mounted with PVA:DABCO. Images were taken using a Leica SP5 confocal microscope and analyzed with FIJI Is Just ImageJ. For quantification of cytoplasmic accumulation of α-syn, the fluorescence intensity in the green channel was corrected using the number of DAns (area green channel/area red channel). The scoring of α-syn-positive DAns was performed by researchers blinded to the experimental conditions.

#### Statistical Analysis

Results are expressed as mean ± SEM. The normal distribution of data was tested using D’Agostino and Pearson omnibus normality test. Student’s *t*-test or one-way ANOVA followed by Tukey’s post hoc test was used for statistical analysis in normal distributions. Statistical analysis for the non-normal distribution data was performed using the Mann–Whitney *U* test or Kruskal–Wallis test. The software used was GraphPad Prism 6, and the value of *p* < 0.05 was considered statistically significant. The number of experiments (*n*) is indicated in the legends to the figures.

## Results and Discussion

### Cationic Carbosilane Dendritic Systems Used in the Studies

**Figure 1:**
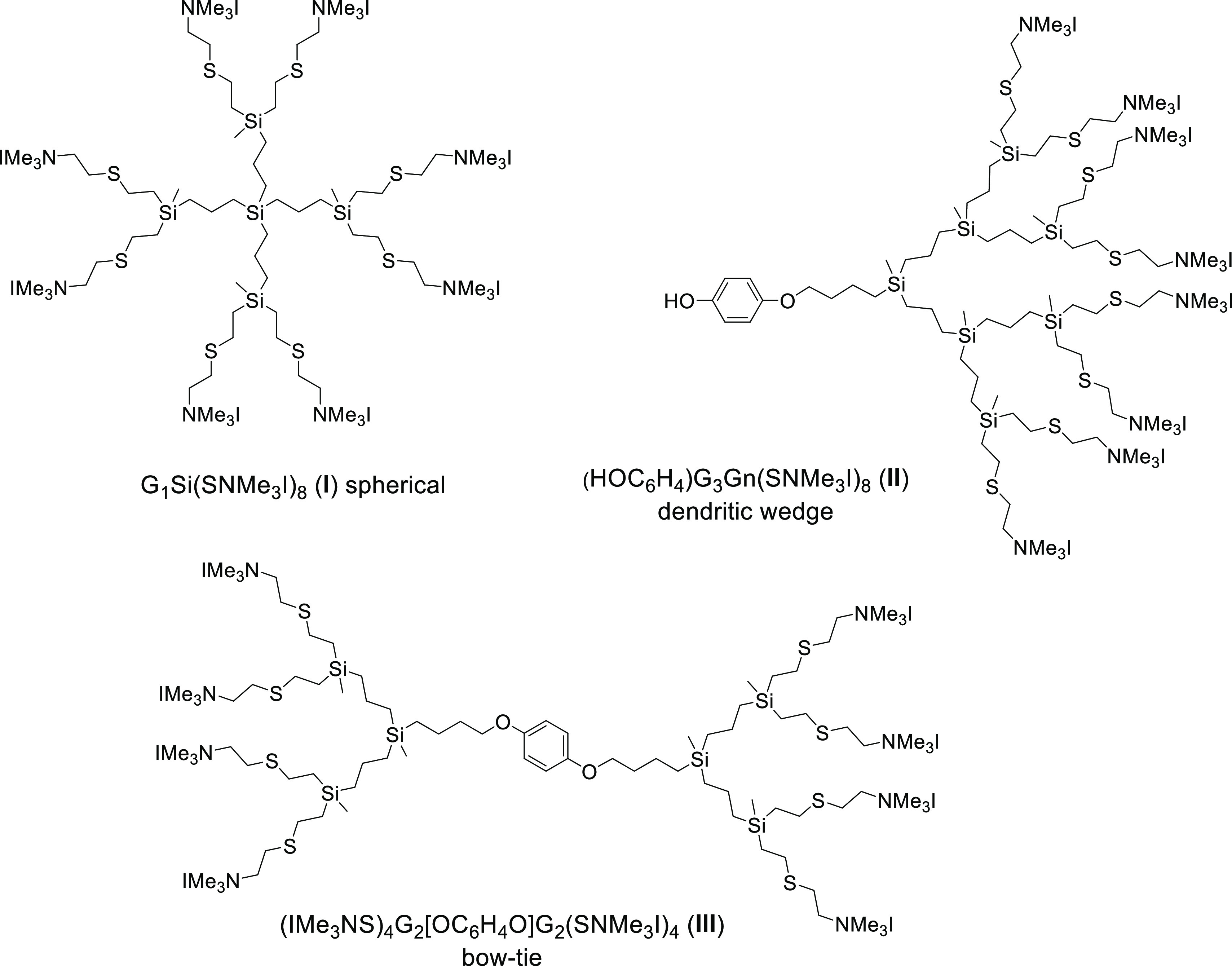
Dendritic structures of G_1_Si(SNMe_3_I)_8_ (**I**) as a spherical dendrimer, (HOC_6_H_4_)G_3_Gn(SNMe_3_I)_8_ (**II**) as a dendron, and (IMe_3_NS)_4_G_2_[OC_6_H_4_O]G_2_(SNMe_3_I)_4_ (**III**) as a bow-tie system.

Previous work in some of our laboratories helped us to identify a strong influence of dendrimer topology on their capacity to interact with amyloid peptides and therefore to inhibit beta-amyloid aggregation in an animal model of type II diabetes.^25^ The morphology (spherical, dendron, or bow-tie) can arrange the same number of peripheral charges differently and therefore modify the interaction with the peptidic domain responsible of the misfolding effect. For this reason, in the current studies, we first set out to investigate the influence of dendritic system topology on α-syn folding. For this purpose, we chose three cationic carbosilane dendritic systems with the same number of charges but different topologies: G_1_Si(SNMe_3_I)_8_ (**I**) as a spherical dendrimer,^41^ HOC_6_H_4_G_3_Gn(SNMe_3_I)_8_ (**II**) as a dendron,^41^ and (IMe_3_NS)_4_G_2_[OC_6_H_4_O]G_2_(SNMe_3_I)_4_ (**III**) as a bow-tie system^25^ (Figure 1).

### Study of Interaction Between α-Syn and the Dendritic System: Biophysical Characterisation

**Figure 2:**
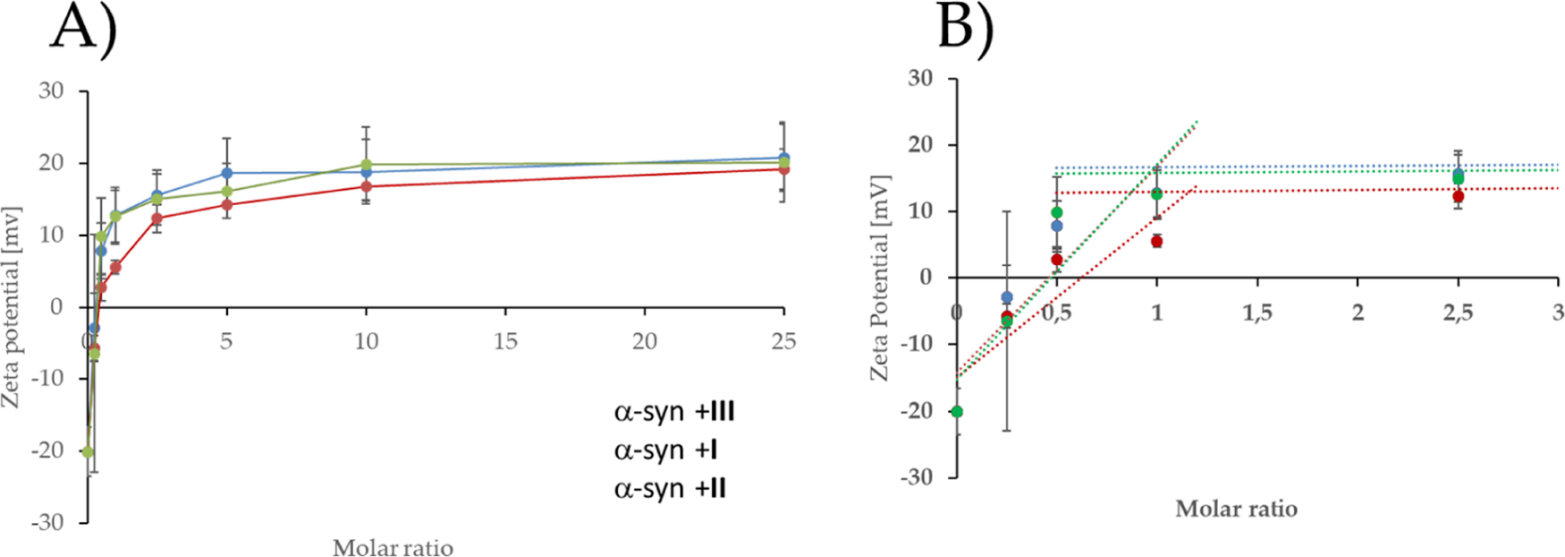
(A) α-Synuclein zeta potential in the presence of carbosilane dendrimers: spherical **I** (red), dendron **II** (green), and bow-tie **III** (blue). (B) Determination of the number of dendritic system molecules attached to α-syn.

The interaction of carbosilane dendrimers and α-syn and their effect on the aggregation status of synthetic peptides of α-syn have been thoroughly analyzed by different biophysical techniques such as zeta potential and circular dichroism.^40^ To ascertain the influence of the system’s topology on such interactions, we first measured changes in zeta potential (directly related to the particle charge environment) to study the interaction between α-syn and different topologies of carbosilane dendrimers. α-Syn is a small acidic protein composed of three well-differentiated domains: (i) a positively charged region called N-terminal lipid-binding α-helix, (ii) a central domain (non-amyloid β component, or NAC) that is involved in the process of aggregation to form cross-β-structures, and (iii) a highly negative and hydrophobic domain known as C-terminal acidic tail.^45^ Due to the predominance of negative charges at pH = 7.4, the α-syn zeta potential value is around −21 mV. However, upon addition of different concentrations of dendritic systems **I–III**, the zeta potential turned positive until reaching a value of ∼20 mV (Figure 2A). Due to the cationic nature of dendritic structures, it is likely that nanoconjugates formed with α-syn have positive charge values independent of the system topology. These results indicate that the topology of the dendrimer system does not significantly influence the zeta potential of the resulting nanoconjugate with α-syn. Moreover, our results also show that nanoconjugates were formed by one molecule of dendrimer/dendron per one α-syn molecule (1:1 molar ratio), in all tested topologies (Figure 2).

**Figure 3:**
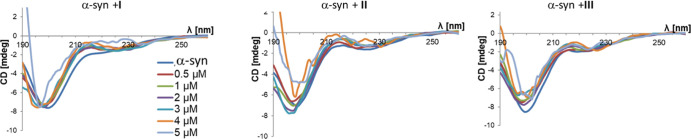
CD spectra of α-synuclein (*c* = 1 μM) alone and in the presence of dendritic systems **I–III**.

**Figure 4:**
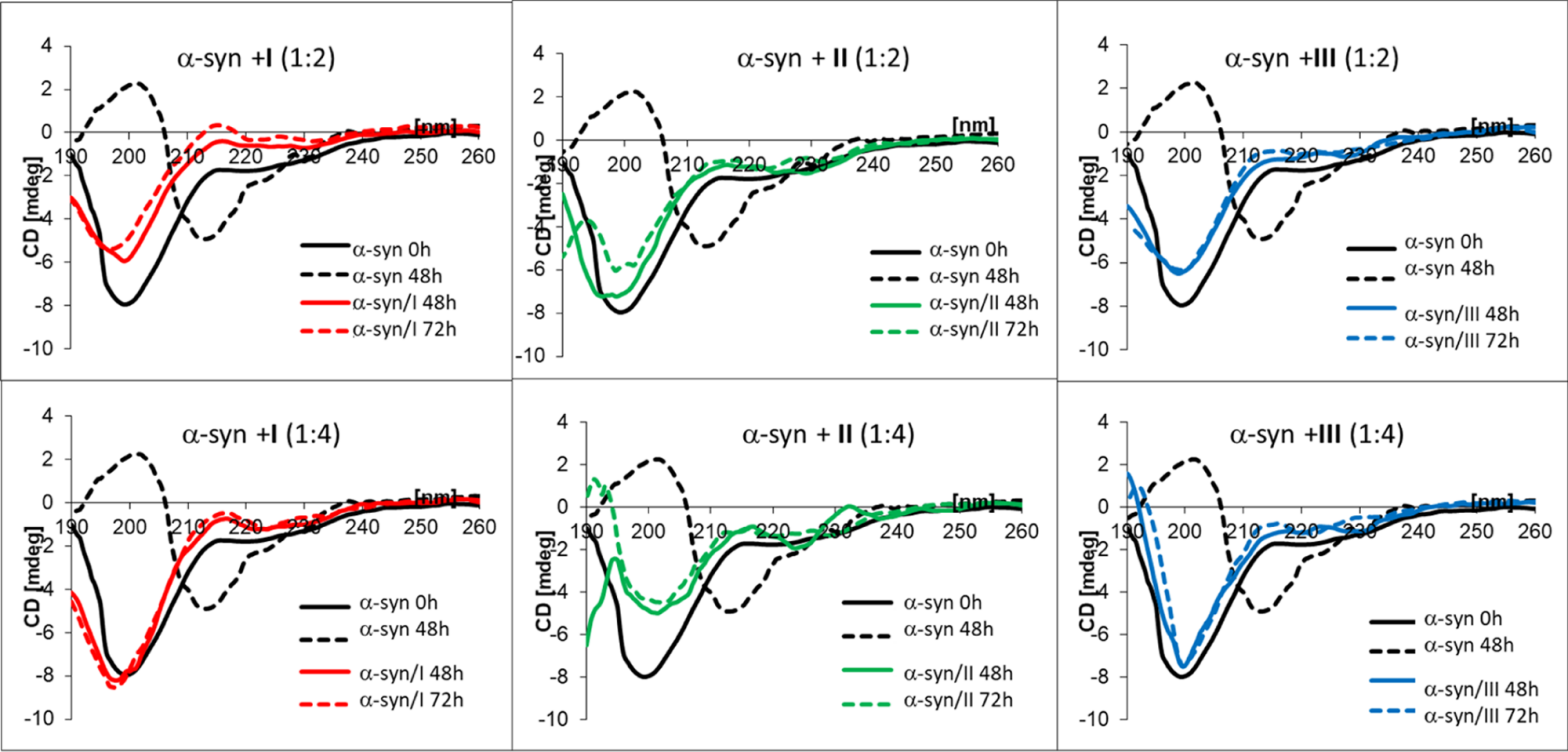
CD spectra of α-synuclein alone and in the presence of dendritic systems **I–III** after 48–72 h of incubation.

We next used CD to analyze conformational changes in the secondary structure of α-syn upon interaction with dendrimers **I–III** under nearly physiological conditions (in solution). The CD spectra of α-syn alone were typical of a substantially unfolded polypeptide chain, with a minimum negative peak characteristic of the random coil observed at 200 nm and a weaker and broader valley centered around 225 nm. Addition of dendrimers **I–III** over a concentration range spanning one order of magnitude (0.5–5 μM) did not cause significant changes in CD spectra except for the largest concentration tested (Figure 3). Taking these results into account, we then evaluated whether dendrimers **I–III** could inhibit the formation of the β structure of α-syn. Upon incubation at 37 °C for 48 h, the CD spectra of α-syn showed a shift to positive values in the range 195–205 nm, indicating the conversion from a disordered structure into the β form (Figure 4). The capacity of dendrimers **I–III** to inhibit this conversion was tested after 48–72 h of incubation in the presence of two different concentrations of dendrimers (α-syn/dendrimer molar ratios of 1:2 or 1:4). All the tested dendrimer topologies exhibited an overall inhibitory activity in the conversion of disordered α-syn into β structure (Figure 4). However, dendron **II** elicited marked changes in α-syn CD spectra, which were more evident at the highest concentration tested, and thus was excluded from further studies. Between the spherical (**I**) and bow-tie (**III**) systems, we chose the latter for downstream experiments in cells due to its relative stronger and more durable inhibiting activity at low concentration (Figure 4) and also because previous tests of the spherical dendrimer **I** in a mouse model of type II diabetes failed to show anti-aggregation activity of the β-amyloid protein.^25^

### Effect of Cationic Carbosilane Dendrimers on the Survival of PD Patient Dopamine Neurons in Culture

**Figure 5:**
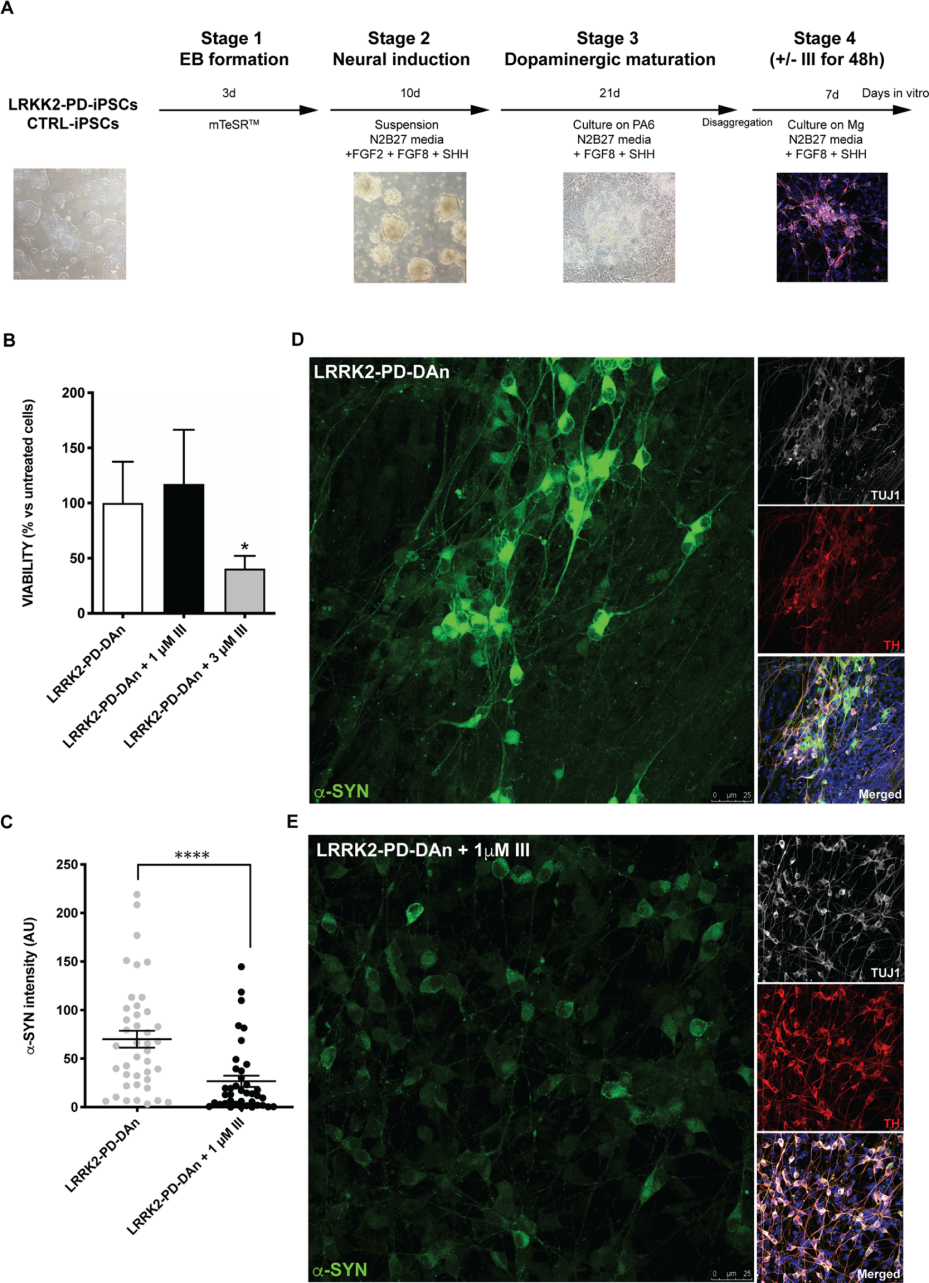
(A) Schematic representation of the differentiation protocol implemented for the generation of DAns from iPSCs generated from two PD patients (LRRK2-PD) and two healthy age-matched controls (CTRL). (B) Analyses of cell viability in an acute exposure (48 h) testing two different doses of dendrimer **III** (1 and 3 μM). Bars represent means with SEM as error bars. (*n* = 4, using 2 LRRK2-PD-DAn lines from two independent experiments for each condition). (C–E) Cytoplasmic accumulation of α-syn in LRRK2-PD-DAns and LRRK2-PD-DAns treated with 1 μM **III** for 48 h. Quantitative analyses of α-syn intensity (*n* = 42–45, using 2 LRRK2-PD-DAn lines from two-independent experiments, and ****p < 0.0001) (C). Representative immunofluorescence images showing diffuse cytoplasmic accumulation of α-syn in LRRK2-PD-DAns (D) and LRRK2-PD-DAns treated with 1 μM **III** for 48 h (E). Scale bars, 25 μm.

Even though the interaction of carbosilane dendrimers and α-syn is well-documented,^40^ as is their capacity to inhibit the aggregation of synthetic peptides of α-syn, to our knowledge, no studies have investigated the effect of carbosilane dendrimers on the endogenous α-syn present in disease-relevant cell types. The relevance of utilizing cell types as similar as possible to those affected by specific diseases when studying pathogenic mechanisms or testing potential therapies is widely recognized.^46,47^ In this respect, reprogramming patients’ somatic cells to pluripotency for the generation of patient- or disease-specific iPSC has emerged as a powerful strategy to produce theoretically unlimited amounts of disease-relevant cell types.^48^ In the case of PD, several laboratories have generated patient-specific iPSC lines from idiopathic and genetic forms of the disease and used them to differentiate DAns that recapitulated key hallmarks of the disease (reviewed in^49−51^). In particular, we have previously shown that DAns differentiated from iPSC obtained from PD patients carrying the G2019S mutation in the *LRRK2* gene (LRRK2-PD) exhibit abnormal accumulation of α-syn^43^ as a consequence of impaired chaperone-mediated autophagy (CMA).^52^ We have also shown that while other LRRK2-PD iPSC-derived brain cells such as astrocytes abnormally accumulate α-syn as well, it is accumulation in DAns that ultimately leads to neurodegeneration.^53^ For these reasons, in the current studies, we set out to test whether cationic carbosilane dendrimers had any effect on the abnormal, disease-related, accumulation of α-syn in LRRK2-PD iPSC-derived DAns. For this purpose, we used iPSC lines representing two LRRK2-PD patients and two healthy individuals as controls, generated and fully characterized in previous work,^43^ and differentiated them into DAns following a robust directed protocol^44^ (see Figure 5A and Experimental section for details). Cationic carbosilane dendrimers (bow-tie system, **III**) were added to DAn cultures at two different concentrations (1 and 3 μM) and incubated for 48 h to first ascertain any effects on DAn survival. Under these conditions, the highest concentration of dendrimer resulted in ∼60% decrease in DAn numbers, as measured by immunostaining for the DAn marker tyrosine hydroxylase (TH), when compared with parallel cultures treated with the vehicle alone (Figure 5B). In contrast, dendrimers were not toxic to DAns when used at 1 μM for 48 h, at least in terms of cell viability (Figure 5B). Indeed, we even observed a slight increase (albeit not statistically significant) in DAn numbers in LRRK2-PD-DAns treated with 1 μM **III** compared to untreated LRRK2-PD-DAns, which could be explained by the dendrimers’ inhibitory effects on DAn α-syn accumulation (see below). Previous work in the context of Alzheimer’s disease showed that several dendrimers were efficient in preventing Aβ-induced cytotoxicity in human neuroblastoma SH-SY5Y cells.^31,54^ Our results so far demonstrate that human DAns derived from PD patient-specific iPSC can safely be cultured, for at least 48 h, in the presence of 1 μM cationic carbosilane dendrimers of bow-tie topology (**III**) and suggest that this treatment could be beneficial for their survival, even though culture times longer than 48 h might be necessary to unambiguously demonstrate the latter.

### Effect of Cationic Carbosilane Dendrimers on the Abnormal Accumulation of α-Syn in DAns from PD Patients

Consistent with previous reports from our laboratories and others,^43,52,55,56^ DAns differentiated from LRRK2-PD iPSCs showed evident cytoplasmic accumulation of α-syn after 30 days of differentiation, which could be quantitatively analyzed from anti-α-syn immunofluorescence intensity (Figure 5C,D). We chose immunolabeling analysis with antibodies against α-syn because this technique has become the standard and most sensitive immunohistochemical method for neuropathological diagnosis of PD.^57^ Treatment of parallel cultures of LRRK2-PD iPSC-derived DAns with 1 μM **III** for the last 48 h of differentiation resulted in a statistically significant decrease in overall α-syn immunoreactivity when compared with untreated cultures (Figure 5C–E).

**Figure 6:**
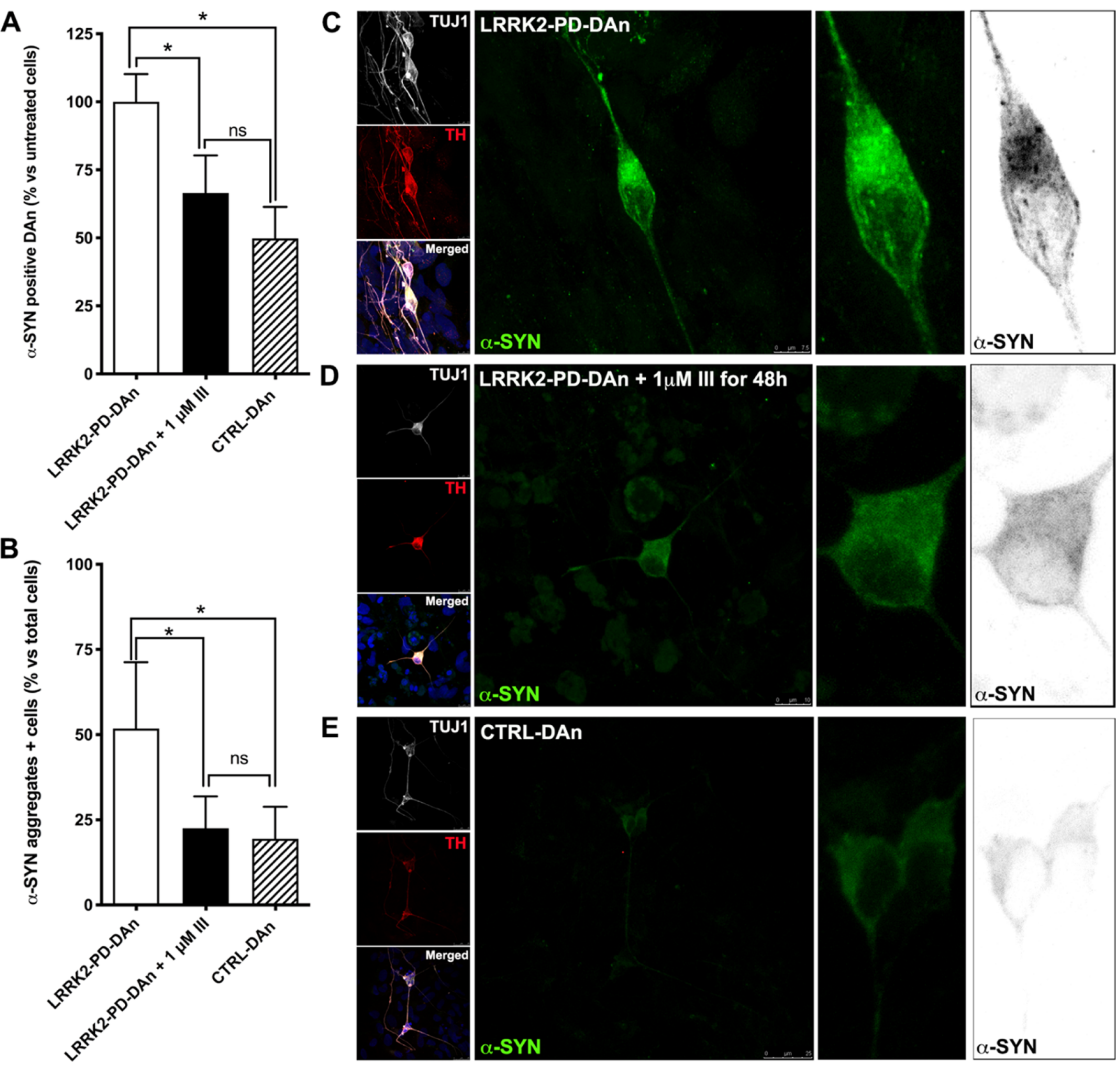
α-Syn accumulation in DAns after 30 days of differentiation. (A,B) Quantitative analyses of α-syn-positive DAn (A) and α-syn aggregates (B). Bars represent means with SEM as error bars. (*n* = 4, using 2 LRRK2-PD-DAn lines and 2 CTRL-DAn lines from two independent experiments). The differences in both, diffuse cytoplasmic α-syn staining and α-syn aggregates accumulation, are statistically significant (*p < 0.05) comparing LRRK2-PD-DAn + 1 μM **III** or CTRL-DAns to untreated LRRK2-PD-DAns. No significant differences (ns) were observed between LRRK2-PD-DAns + 1 μM **III** and CTRL-DAns. (C–E) Representative immunofluorescence images showing α-syn aggregates accumulation in LRRK2-PD-DAns; scale bar, 7.5 μm (C), LRRK2-PD-DAns treated with 1 μM **III** for 48 h; scale bar, 10 μm (D), and CTRL-DAns; scale bar, 25 μm (E).

Dendrimer effects were also evident when we quantified the percentage of DAns that stained positive for α-syn. Under our differentiation and culture conditions, DAns from LRKK2-PD patients accumulate α-syn in their cytoplasm (and are thus scored as α-syn-positive DAns) at approximately twice the frequency when compared with DAns from control individuals (Figure 6A). Using DAns differentiated from two independent LRKK2-PD patients, we found that treatment with 1 μM **III** for 48 h was sufficient to reduce the percentage of α-syn-positive DAns to values not statistically different from those measured in control DAns (Figure 6A).

We next analyzed the capacity of dendrimers to reduce the intracellular accumulation of α-syn aggregates. Imaging by confocal microscopy of anti-α-syn immunostained DAns identifies α-syn aggregates as distinct puncta accumulated in the cell body.^58^ In our experiments, the percentage of LRKK2-PD DAns containing α-syn puncta in the cell body was significantly higher than that of control DAns (51.8 ± 8.7% *vs.* 19.4 ± 4.7%, *n* = 5 and 4 independent differentiations using two iPSC lines per condition; Figure 6B–E). These results are in line with previous evidence^43,52,53^ and highlight the advantage of using patient-specific iPSC-based models compared to other experimental systems where additional manipulations, such as treatment with exogenous α-syn fibrils or rotenone-induced α-syn fibrillation, are required.^22,40^ Importantly, incubation of LRKK2-PD DAns with 1 μM **III** for 48 h reduced the percentage of cells displaying α-syn puncta to values similar to those shown by control DAns (Figure 6B–E). These results suggest that cationic carbosilane dendrimers of bow-tie topology (**III**), when used at non-toxic concentrations, are internalized by PD patients’ DAns and directly interact with α-syn aggregates, probably helping to solubilize them into smaller aggregates that would be more easily degraded.

## Conclusions

In summary, in this work, we demonstrate the effectiveness of cationic carbosilane dendrimers toward key PD-related cell phenotypes using an experimental system based on disease-relevant cell types from PD patients. We further show the influence of dendritic topology on the interaction with α-syn and, therefore, on their ability to prevent the formation of α-syn aggregates. Bow-tie dendrimers showed good performance and were well tolerated by DAns, a cell type especially susceptible to toxic insults.^59^ The development of new therapeutic approaches for PD requires the use of appropriate experimental conditions mimicking the neurodegeneration that takes place in patients. In this sense, iPSC technology^60^ has opened unprecedented opportunities for the generation of genuinely human models of disease.^48^ Dendrimers have been shown to interfere with the formation of amyloid fibrillary structures typically related with the onset and progression of protein misfolding diseases such as Alzheimer’s disease^28,29,54^ and prion diseases.^61,62^ Thus, dendrimers as anti-neurodegenerative agents may have application in PD therapy in which disease progression is closely correlated to brain accumulation of insoluble α-syn. Numerous previous studies have explored the usefulness of dendritic systems to prevent and/or rescue α-syn aggregation in a variety of cell-free and cell-based experimental models of PD.^21,22,24,26,27^ Our results lend additional support to this line of study and, importantly, validate the development of dendrimer-based anti-PD therapies using DAns, the cell type most relevant for the disease, obtained from PD patients. At the cellular level, our results confirmed a significant decrease in cytoplasmic levels of α-syn and in α-syn aggregates in DAns from LRRK2-PD-iPSC treated during 48 h with non-cytotoxic concentrations of bow-tie dendrimer **III**. We believe that the results from our studies will help develop safe and efficient therapies for delaying or even halting PD progression.
